# Myocardial sympathetic distal axon loss in subjects with Lewy pathology in three autopsy cohorts

**DOI:** 10.1007/s00401-025-02918-y

**Published:** 2025-08-01

**Authors:** Ville Kivistö, Benjamin Englert, Jarno Tuimala, Eloise Kok, Henri Puttonen, Anna Raunio, Pekka J. Karhunen, Maria K. Lehtinen, Per Borghammer, Ella Ahvenainen, Kia Colangelo, Sara Savola, Maarit Tanskanen, Karri Kaivola, Pentti J. Tienari, Darshan Kumar, Anders Paetau, Olli Tynninen, Mikko I. Mäyränpää, Tuomo Polvikoski, Liisa Myllykangas

**Affiliations:** 1 Department of Pathology, University of Helsinki, 00014 Helsinki, Finland; 2 HUS Diagnostic Center at Helsinki University Hospital, 00029 Helsinki, Finland; 3 Faculty of Medicine and Health Technology, Tampere University, 33100 Tampere, Finland; 4 Finnish Cardiovascular Research Center, 33014 Tampere, Finland; 5 Fimlab Laboratories Ltd., Wellbeing Services County of Pirkanmaa, 33520 Tampere, Finland; 6 Department of Pathology, Boston Children’s Hospital and Harvard Medical School, Boston, MA 02115 USA; 7 Institute of Clinical Medicine, Aarhus University, 8200 Aarhus, Denmark; 8 Translational Immunology, Research Programs Unit, University of Helsinki, 00014 Helsinki, Finland; 9 Brain Center, Neurology, HUS, Helsinki University Hospital, 00014 Helsinki, Finland; 10 Aiforia Technologies Oyj., 00150 Helsinki, Finland; 11 Newcastle University Translational and Clinical Research Institute, University of Newcastle, Newcastle Upon Tyne, NE1 7RU UK

**Keywords:** Lewy body disease, Image recognition tool, Neuropathology, Post-mortem, Neurodegeneration, Myocardium

## Abstract

Cardiac manifestations are associated with Lewy body disease, but studies addressing the underlying histopathological mechanisms at the myocardial level are sparse. Here, we generated an artificial intelligence-based algorithm to quantify tyrosine hydroxylase (TH)-immunoreactive sympathetic distal axons at the myocardial level. This novel tool was applied to septal samples of the Vantaa 85 + study (*n* = 138), which is a population-based autopsy study representing all subjects aged 85 years or older living in the city of Vantaa (southern Finland) in 1991. In addition, the tool was applied to left ventricle samples of the Helsinki Biobank (*n* = 87) and the forensic Tampere Sudden Death Study (TSDS, *n* = 127). The level of myocardial TH reactivity was compared between subjects with and without Lewy pathology in the central nervous system in all datasets. In the Vantaa 85 + study, TH reactivity was also compared between subjects with caudo-rostral and amygdala-based subtypes, and potential confounding factors (age at death, sex, myocardial infarction, senile systemic amyloidosis, and diabetes medication) were controlled for using multiple linear regression models. Presence of Lewy pathology was strongly associated with loss of TH reactivity at the myocardial level in all three autopsy cohorts (Vantaa 85 + *p* = 0.001, Helsinki Biobank *p* < 0.001, TSDS *p* < 0.001)). In the Vantaa 85 + study, the caudo-rostral subtype (*p* < 0.001), but not the amygdala-based subtype (*p* = 0.60), was associated with myocardial denervation/dysfunction, and this association was independent of other known causes of sympathetic denervation/dysfunction. Caudo-rostral subtype and myocardial infarction were the strongest predictors of myocardial sympathetic denervation/dysfunction in the oldest-old population (Vantaa 85 +). In conclusion, our results show that Lewy pathology in the central nervous system, and particularly its caudo-rostral subtype, is strongly associated with loss of sympathetic distal axons at the myocardial level. We also provide evidence that the caudo-rostral subtype is one of the strongest predictors of myocardial sympathetic denervation/dysfunction in the oldest-old population.

**Supplementary Information:**

The online version contains supplementary material available at 10.1007/s00401-025-02918-y.

## Introduction

Lewy body disease (LBD) refers to a neurodegenerative disease characterized by accumulation of misfolded alpha-synuclein (α-syn), which clinically manifests as Parkinson’s disease (PD) or dementia with Lewy bodies (DLB). The pathological hallmark of LBD is Lewy pathology, consisting of Lewy bodies and neurites, found in neurons [23, 33].

Increasing evidence supports multiple subtypes of LBD. A common framework categorizes cases into body-first and brain-first subtypes informed by the distribution of pathology in the central (CNS) and peripheral nervous systems and the order in which symptoms present [3–5]. Further subdivisions have also been proposed based on histological data analyzed with the Subtype and Stage Inference (SuStaIn) model [2, 21]. In accordance with the body-first/brain-first framework, we have previously described the existence of two common LBD subtypes in the Vantaa 85 + study based on pathological data, which we have termed caudo-rostral and amygdala-based subtypes [29].

It is widely acknowledged that LBD affects the whole nervous system, including the peripheral nervous system. In particular, cardiac manifestations of LBD have become a point of interest. For example, it has been reported that LBD patients have an increased risk of sudden cardiac death compared to patients with other types of dementia [16]. In addition, patients with DLB were reportedly more likely to receive pacemakers to treat sick sinus syndrome than patients with Alzheimer’s disease (AD) [12], and some studies have suggested that both PD and DLB are associated with an increased risk of atrial fibrillation [7, 35]. Heart rate variability during sleep has also been shown to be associated with PD and multiple system atrophy (MSA) [25], another form of synucleinopathy characterized by accumulation of oligodendroglial α-syn inclusions [40].

Histologically, α-syn aggregates are commonly found in cardiac tissue and sympathetic ganglia sections of patients with LBD [1, 15, 36]. A previous study by Orimo and coworkers found cardiac α-syn and degeneration of epicardial sympathetic nerve fascicles in PD and incidental LBD, i.e., Lewy pathology present without clinical signs of parkinsonism, but not in controls. Furthermore, cases with more severe epicardial sympathetic degeneration had less severe α-syn pathology in the heart. Based on these findings, it was proposed that cardiac α-syn accumulates in sympathetic axons and as the axons degenerate, the associated α-syn disappears as well [26]. Cardiac sympathetic degeneration in large epicardial nerve fascicles of LBD patients has been replicated in several semi-quantitative and one quantitative study [1, 22, 36], but literature describing the more distal innervation in the myocardium is sparse [8, 17].

Cardiac sympathetic dysfunction in LBD has been widely demonstrated in imaging studies. In a study by Goldstein et al. [11], 20 of 29 patients with PD showed low interventricular septal 6-[^18^F]fluorodopamine–derived radioactivity, indicating sympathetic dysfunction. In comparison, the sympathetic innervation in MSA seems relatively preserved [10]. Abnormal cardiac sympathetic innervation in LBD patients has also been demonstrated using ^123^iodine-meta-iodobenzylguanidine (MIBG) myocardial scintigraphy, and this finding correlates with histological loss of cardiac sympathetic innervation [22, 36]. Cardiac MIBG can also be used to distinguish DLB from other dementias [22, 38, 41]. A recent MIBG study, which used isolated REM-sleep behavioral disorder as a clinical marker for prodromal body-first cases, provided support for the hypothesis of body-first LBD cases showing early cardiac sympathetic dysfunction [13], but histopathological evidence is lacking. In addition, it is unclear how specific the sympathetic denervation is to LBD as senile systemic amyloidosis, myocardial infarction, and diabetes have also been associated with abnormal MIBG scans [9, 31, 34].

We hypothesized that an artificial intelligence-based algorithm quantifying tyrosine hydroxylase (TH)-immunoreactive distal axons at the myocardial level would provide a sensitive method to study the possible myocardial sympathetic denervation/dysfunction in subjects with different forms of synucleinopathy. By applying this novel tool to three independent autopsy cohorts, we show that the presence of CNS Lewy pathology is strongly associated with loss of distal sympathetic axons at the myocardial level. Furthermore, we provide the first histopathological evidence in support of the body-first (caudo-rostral) subtype of LBD being prone to earlier and more severe myocardial denervation/dysfunction, occurring independently of other known causes of sympathetic denervation/dysfunction.

## Materials and methods

### Cohort descriptions

For the present study, we included individuals from three independent autopsy cohorts.

Vantaa 85 + is a population-based study based on all individuals 85 years old or older living in the city of Vantaa (southern Finland) on April 1, 1991 (*n* = 601). A comprehensive description of the cohort has been published previously [27–29, 37]. General and neuropathological autopsy was performed whenever possible, and eventually 304 (51%) underwent consented general and neuropathological post-mortem examination. The extent of post-mortem examination was not influenced by the cause of death. Of these 304 subjects, 139 were found to have Lewy pathology in the CNS [30]. In addition, the LBD subtype of caudo-rostral or amygdala-based as well as the DLB consortium classification has been determined [23, 24, 29]. Assessments of myocardial infarction, senile systemic amyloidosis, and diabetes medication have been described previously [37]. Heart samples were collected from the septum in a standardized manner regardless of macroscopic pathology for 138 of the 304 neuropathologically examined subjects, comprising the subset used in the present study. Supplementary Fig. 1 illustrates the inclusion protocol of Vantaa 85 + subjects in the present study, and Supplementary Table 1 presents the demographic details of the included subjects. Age at death ranged from 86 to 106 years in the Vantaa 85 + cohort.

The Helsinki Biobank collects various tissue samples, including samples acquired as part of routine autopsies performed at the Helsinki University Hospital. For the present study, we collected left ventricle and brainstem samples from cases neuropathologically diagnosed with LBD, MSA, AD, progressive supranuclear palsy (PSP), or with no neurodegenerative disease at autopsy. Subjects with uncertainty about the neuropathological diagnosis were excluded. AD neuropathological changes were accepted as co-pathology in LBD subjects. As Lewy pathology is sometimes seen as a secondary pathology in different neurodegenerative diseases, we also sought to stain the brainstem of all Helsinki Biobank subjects for α-syn. Cases with Lewy pathology present in the brainstem (primarily the substantia nigra, but pons and medulla were stained in 2 subjects without substantia nigra available) or brainstem samples missing without prior neuropathological LBD diagnosis were excluded (*n* = 29). However, as concurrent Lewy pathology is common in MSA, we did not exclude MSA cases with Lewy pathology in the brainstem (*n* = 6) or missing brainstem samples (*n* = 2). Applying these criteria, we included 54 cases neuropathologically diagnosed with LBD, whose DLB consortium classification was defined [23, 24], 13 cases neuropathologically diagnosed with MSA, and 20 cases that had not been neuropathologically diagnosed with LBD for a total of 87 subjects. Age at death ranged from 44 to 95 in the Helsinki Biobank cohort. Further demographic and neuropathological details of the Helsinki Biobank subjects are presented in Supplementary Tables 2 and 3.

The Tampere Sudden Death Study (TSDS) is a forensic autopsy cohort consisting of 700 cases who died outside hospital in the Tampere region (southern Finland) in 2010–2015, representing approximately 20% of deaths in the region during the timeframe. For this study, we acquired left ventricle samples from 37 cases previously shown to have Lewy pathology, 86 age and sex-matched controls without Lewy pathology, and 4 cases with MSA-type pathology [18], for a total of 127 subjects. Ages at death ranged from 42 to 90. None of the MSA cases in the TSDS cohort displayed concurrent Lewy pathology. DLB consortium classification was estimated by assessing Lewy pathology in brainstem, hippocampus, and frontal cortex [24]. The demographic details of the TSDS subjects are presented in Supplementary Table 4.

In all cohorts, cases with CNS Lewy pathology formed the “LP-positive” group, and those without CNS Lewy pathology formed the “LP-negative” group. MSA cases were considered separately as the “MSA” group. Detailed explanations of the variables used in this study are presented in Supplementary Table 5 for all the cohorts.

### Classification of LBD subtypes in the Vantaa 85 + cohort

LBD cases in the Vantaa 85 + cohort were determined to be either caudo-rostral or amygdala-based in the original study by Raunio et al. [29] according to a systematic anatomical scoring of Lewy pathology in the CNS. The subtype was determined by the profile of Lewy pathology scores in the analyzed areas (examples shown in Supplementary Figs. 2, 3). Subtyping was further supported by an unsupervised K-means clustering analysis [29]. These subtypes have been suggested to correspond to the body-first and brain-first subtypes as described by Borghammer et al. [5] and similar profiles of pathology have been reported in other autopsy cohorts [2, 5]. The guidelines used by Raunio et al. [29] were the following:

- Caudo-rostral was defined by the strongest Lewy pathology at the medulla and brainstem regions with a declining gradient of Lewy pathology toward more rostral regions (amygdala, limbic, cortical). The score at the medulla and brainstem areas was at least one score higher than in the limbic areas.
- Amygdala-based was defined by the strongest pathology in the amygdala and/or the limbic regions with a declining gradient toward more caudal (brainstem, medulla, and spinal cord) or cortical regions. The score at the amygdala/limbic regions was at least one score higher than in the neighboring cortical or brainstem areas.
- One subject could not be categorized as every brain region was scored as very severe. (No septal sample available, not included in the present study)

Additionally, 8 cases with a septal sample available were originally determined as no LBD by Raunio et al. [29] but were shown to harbor Lewy pathology only in the olfactory bulb/peduncle by Kok et al.[19]. In the present study, these 8 cases are included in the LP-positive group as amygdala-based cases as the amygdala-based (brain-first) subtype is associated with olfactory bulb pathology [6, 19].

### Immunohistochemistry

To quantify sympathetic innervation, all heart samples were immunohistochemically stained using an anti-TH antibody (Thermo Fisher Scientific Cat# MA5-32984, RRID: AB_2802618). Furthermore, all heart samples and brainstem samples of the Helsinki Biobank cohort were stained using an anti-α-syn antibody (Millipore Cat# MABN389, RRID: AB_2716647). To study colocalization of TH reactivity with neuronal/axonal markers, immunohistochemistry with an antibody against protein gene product (PGP9.5, Abcam, Cat# ab108986, RRID: AB_10891773) and phosphorylated neurofilament (SMI-31, Nordic Biosite, Cat# SMI-31R) was carried out on a small subset of samples. In the Vantaa 85 + and TSDS cohorts, α-syn staining of the CNS samples had been performed previously [18, 29].

Tissue samples were fixed in formalin and embedded in paraffin after sampling. The tissue fixation protocol was similar for all cohorts, with formalin fixation times being at most two weeks. In preparation for immunohistochemical staining, samples were cut into 10 µm thick sections for PGP9.5 staining and 4 µm thick sections for other IHC stainings, placed on glass slides, and dried at + 37 ℃ overnight. The samples were then heated at + 56 ℃ for two hours and went through deparaffinization and rehydration using consecutive xylene baths followed by a series of ethanol baths with decreasing ethanol content. Samples were then incubated in a pH9 buffer (PGP9.5 antibody) or pH6 buffer (other antibodies) (Dako Target retrieval solution K2005) at + 95 ℃ for 20 min, washed, and treated in formic acid for 5 min.

Antibody treatment was done using a machine (Labvision Autostainer 480) with the EnVision FLEX -kit (K8002). The anti-α-syn antibody was diluted to 1:4000 with Dako antibody diluent (S2022), while the anti-TH antibody was diluted to 1:400, the anti-SMI-31 antibody to 1:2000 and the anti-PGP9.5 antibody to 1:250 with the same antibody diluent. Negative controls were made to check for possible false positivity, which was not seen.

### Image recognition algorithm and sample analysis

To analyze the sympathetic innervation in the heart samples, we developed an image recognition algorithm based on an artificial intelligence tool (Aiforia^®^) that quantitatively measures the area of TH-reactive staining in digitalised cardiac tissue sections. The algorithm also measures the tissue area and separates larger nerve fascicles from other tissue. The algorithm was trained using samples from all three cohorts, and its performance was validated by comparing its results against annotations made by three board-certified pathologists (L.M., H.P., M.M.). Detailed description of the training and validation of the algorithm are presented in the Supplementary material under section “Image recognition algorithm training and validation”.

From each sample, we analyzed a 5 mm wide rectangular area drawn on the Aiforia Create platform spanning the myocardium from endocardium to endocardium in the septum samples and epicardium to endocardium in the left ventricle samples, excluding any epicardial fat tissue. To measure the myocardial distal axon TH reactivity, we calculated the area of TH-reactive staining in the analyzed tissue area. Large nerve fascicles (diameter > 20 µm) were excluded from the tissue area (TH-reactive staining inside these fascicles was also excluded). We then calculated the relative TH reactivity by dividing the remaining TH-reactive staining area by the remaining tissue area. The method thus controls for possible variation in ventricular wall thickness.

All available α-syn IHC-stained heart samples were manually analyzed and semiquantitatively scored for α-syn pathology on a scale of 0–3 (0 = no pathology; 1 = focal, sparse pathology; 2 = local, moderate pathology; 3 = widespread, severe pathology).

### Statistical analyses

The two-sided Mann–Whitney U test was used to test for differences in group distributions regarding relative myocardial TH reactivity as the data did not follow a normal distribution. Multiple linear regression was used to model the effects of different explanatory variables with relative myocardial TH reactivity as the dependent variable. In the linear regression models, a Box–Cox transformation was performed on the relative myocardial TH reactivity to alleviate heteroscedasticity and normalize the data. Statistical tests were performed using the stats package in RStudio version 2024.12.1 with R version 4.4.3. *P *values were not adjusted for multiplicity. *P* < 0.05 was considered significant.

### Ethical considerations

Local ethical committees approved each of the three studies, followed by research permission by the Helsinki University Hospital (Vantaa 85 + and the Helsinki Biobank) and Tampere University (TSDS). In the Vantaa 85 + study, each participant and/or their relatives have given informed consent for the study and relatives have given written consent for autopsy. In accordance with the Finnish national ethical standards and the Act on the Medical Use of Human Organs, Tissues and Cells (101/2001), which allow using post-mortem samples collected as part of medical or forensic autopsies in research, informed consent was not obtained (or required) for the Helsinki Biobank and TSDS cohorts.

## Results

### Image recognition algorithm validation results

**Fig. 1:**
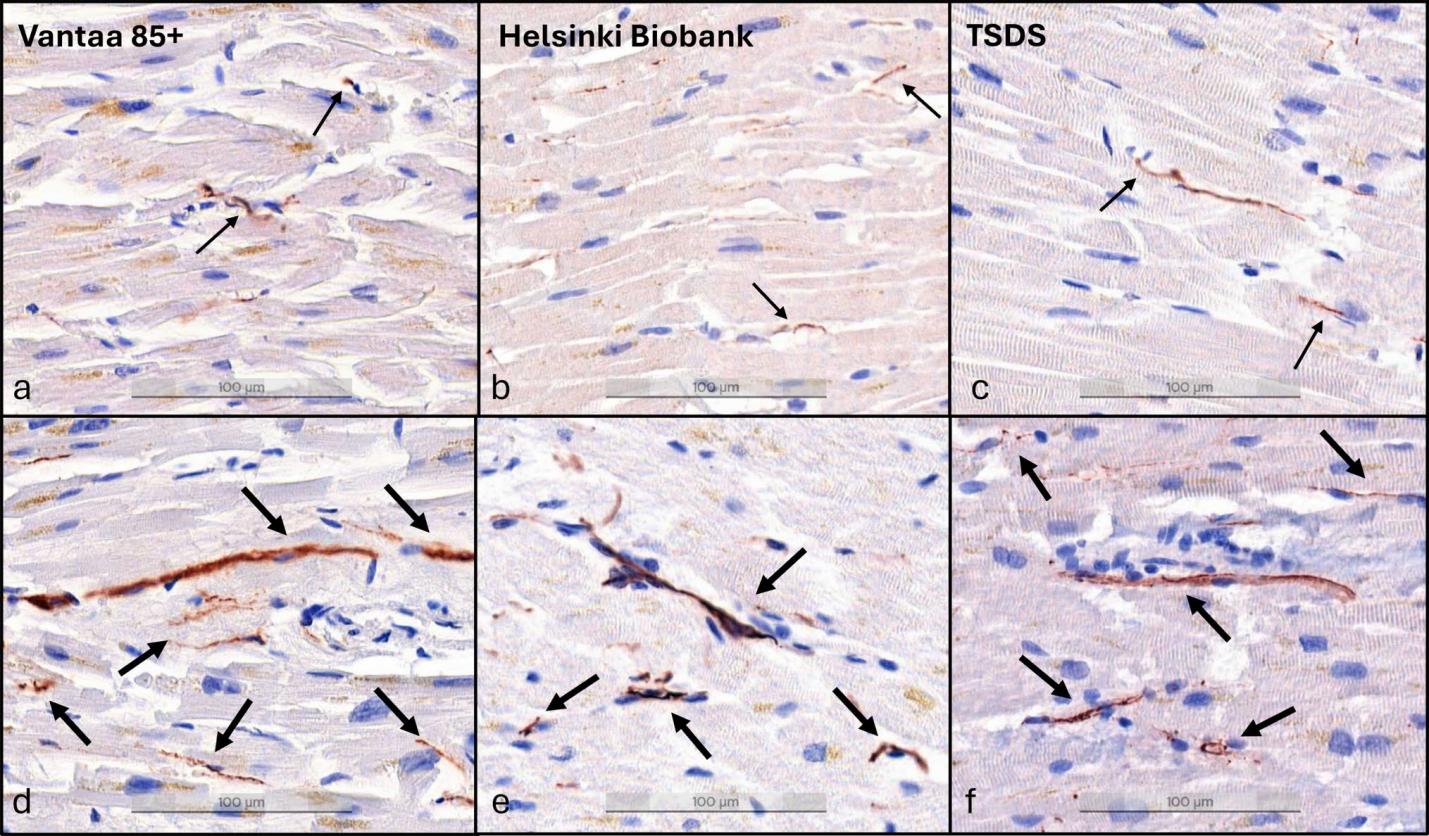
Example myocardial samples with decreased and preserved TH reactivity. **a-c**: subjects with decreased TH reactivity (thin arrows). **d-f**: subjects with preserved TH reactivity (thick arrows). Pictures taken at 200 × magnification. Scale bar shown at the bottom of each image. *TH* tyrosine hydroxylase

The image recognition algorithm displayed good performance in quantifying the TH-stained area with an average sensitivity and precision of 78.90% and 83.96%. Recognition of non-nerve fascicle tissue was also reliable, with an average sensitivity and precision of 99.21% and 98.23%. A small subset of the samples was stained with the neuronal/axonal markers SMI-31 and PGP9.5 to confirm that the TH-positive staining was representative of sympathetic neuronal structures (Supplementary Figs. 4, 5). Examples of the algorithm detecting TH-positive staining and non-nerve fascicle tissue are shown in Supplementary Figs. 6, 7, and examples of samples with decreased and preserved TH reactivity are shown in Fig. 1. More details and parameters of the validation are presented in Supplementary material and Supplementary Table 6.

### Association of cardiac TH reactivity with CNS Lewy pathology and MSA

**Fig. 2:**
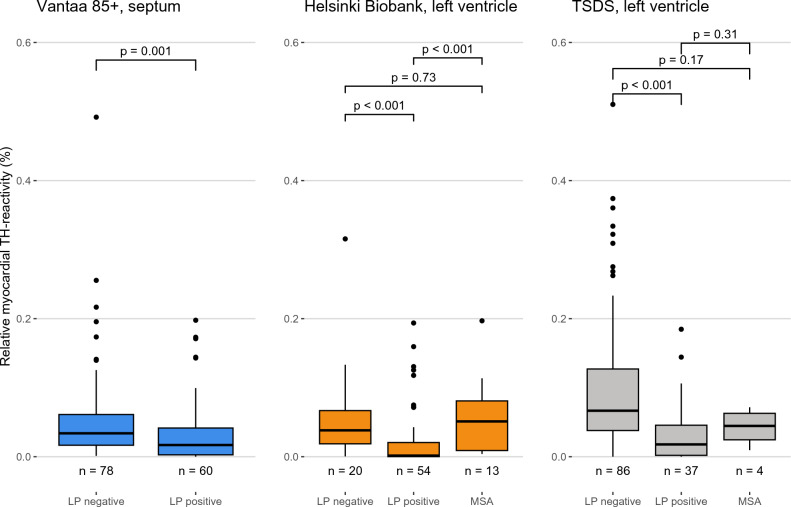
Relationship between relative myocardial TH reactivity and CNS Lewy pathology and MSA. Relative myocardial TH reactivity is defined as the area of TH-positive staining divided by analyzed tissue area, excluding the tissue and TH-positive staining in larger nerve fascicles (diameter > 20 µm). Box-and-whiskers plots with median, 1st to 3rd interquartile range, and minimum and maximum. Outliers shown as dots. *P* values for group differences calculated using Mann–Whitney U test. *TH* tyrosine hydroxylase, *CNS* central nervous system, *LP positive* CNS Lewy pathology-positive, *LP negative* CNS Lewy pathology-negative, *MSA* multiple system atrophy

We first studied the association of myocardial TH reactivity with LBD in all three cohorts by stratifying cases according to the presence or absence of Lewy pathology in the CNS (LP-positive vs. LP-negative). In each cohort, the LP-positive group had statistically significantly lower TH reactivity (*P* ≤ 0.001) compared to the LP-negative group (Median TH reactivity [interquartile range] for LP-positive and negative groups: 0.017% [0.039] and 0.034% [0.044] in Vantaa 85 + ; 0.002% [0.020] and 0.038% [0.048] in Helsinki Biobank; 0.018% [0.044] and 0.067% [0.089] in TSDS) (Fig. 2).

We also analyzed the difference between the LP groups and the MSA group in the Helsinki Biobank and TSDS cohorts (the Vantaa 85 + study did not include any MSA cases with septal samples available). MSA cases did not significantly differ from the LP-negative group in either cohort (Median TH reactivity [interquartile range] in MSA cases: 0.051% [0.072] in Helsinki Biobank; 0.045% [0.038] in TSDS). In the Helsinki Biobank cohort, the LP-positive group had lower TH reactivity compared to the MSA group (*P* < 0.001), and a similar but statistically non-significant trend was seen in the TSDS cohort (Fig. 2). Means and standard deviations of these groups are presented in Supplementary Table 7.

### Association of cardiac TH reactivity and LBD subtypes

**Fig. 3:**
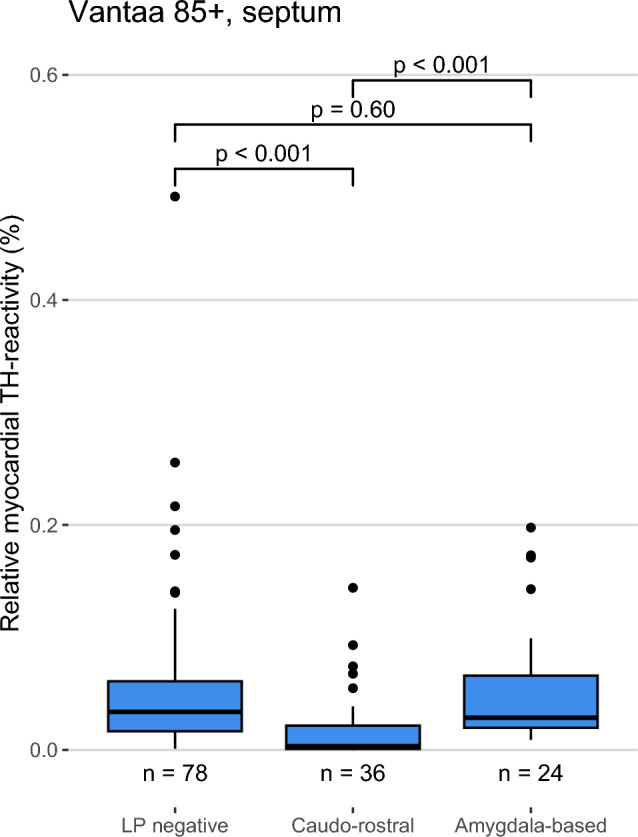
Relationship between the LBD subtypes and relative myocardial TH reactivity in Vantaa 85 + subjects. Relative myocardial TH reactivity is defined as the area of TH-positive staining divided by analyzed tissue area, excluding the tissue and TH-positive staining in larger nerve fascicles (diameter > 20 µm). Box-and-whiskers plot with median, 1st to 3rd interquartile range, and minimum and maximum. Outliers shown as dots. *P* values calculated using Mann–Whitney U test. *LBD* Lewy body disease, *TH* tyrosine hydroxylase, *CNS* central nervous system, *LP negative* CNS Lewy pathology-negative

Previous imaging studies have suggested that the body-first (caudo-rostral) LBD subtype is associated with earlier cardiac sympathetic impairment [13, 14]. To assess the effect of the subtypes, we compared the LP-negative group to the caudo-rostral and amygdala-based groups in the Vantaa 85 + cohort. We found that the caudo-rostral subtype was statistically significantly associated with lower TH reactivity compared to the LP-negative group and the amygdala-based group, while the amygdala-based subtype showed no difference to the LP-negative group (Median TH reactivity [interquartile range] in caudo-rostral and amygdala-based groups: 0.003% [0.021] and 0.029% [0.046]) (Fig. 3). Means and standard deviations are presented in Supplementary Table 8.

We next performed linear regression analyses to further assess whether the effect of caudo-rostral LBD subtype in the Vantaa 85 + subjects was independent of age, sex, or other factors known to be associated with cardiac sympathetic denervation/dysfunction (Table 1). A negative association with the caudo-rostral subtype persisted in all four tested models and showed the strongest statistical significance and largest effect estimate out of all variables (*P* < 0.001), while the amygdala-based subtype showed no association in any model. Myocardial infarction was also statistically significantly associated with lower TH reactivity in all models in which it was included (*P* < 0.001); however, its effect size estimate was not as large as the caudo-rostral subtype. Age, sex, diabetes medication, and senile systemic amyloidosis were not statistically significantly associated with TH reactivity in any of the four models.

**Table 1:** Multiple linear regression models of relative myocardial TH reactivity in Vantaa 85 + subjects, septum

	Model 1	Model 2	Model 3	Model 4
Constant	−1.959 [−4.817, 0.900]	−2.194 [−4.945, 0.558]	−1.779 [−4.560, 1.002]	−1.396 [−4.238, 1.447]
Age at death	−0.006 [−0.036, 0.024]	−0.002 [−0.031, 0.027]	−0.006 [−0.036, 0.023]	−0.010 [−0.040, 0.020]
Female sex	0.003 [−0.322, 0.328]	0.042 [−0.282, 0.365]	0.086 [−0.240, 0.413]	0.028 [−0.321, 0.378]
Caudo-rostral subtype	−0.859*** [−1.134, −0.585]	−0.895*** [−1.166, −0.625]	−0.879*** [−1.149, −0.610]	−0.828*** [−1.105, −0.551]
Amygdala-based subtype	0.101 [−0.204, 0.406]	0.051 [−0.245, 0.348]	0.034 [−0.261, 0.329]	0.012 [−0.299, 0.322]
Myocardial infarction	NA	−0.451*** [−0.682, −0.221]	−0.440*** [−0.670, −0.211]	−0.523*** [−0.767, −0.279]
Diabetes medication	NA	NA	−0.221 [−0.493, 0.051]	−0.220 [−0.494, 0.055]
Senile systemic amyloidosis	NA	NA	NA	0.106 [−0.164, 0.375]
Num.Obs	138	129	129	120

Multiple linear regression models for relative myocardial TH reactivity as percentage in septum samples of Vantaa 85 + subjects. The table shows the coefficient estimate and its statistical significance as stars and 95% confidence interval inside square brackets. A Box–Cox transformation was used on the dependent variable to normalize the data and alleviate heteroscedasticity. The estimates and confidence intervals are shown in the transformed form and do not represent the estimate as change in percentage, as Box–Cox does not allow for back-transformation of the estimate and confidence intervals. The number of subjects without missing data for the independent variables are shown at the bottom for each model

*TH* tyrosine hydroxylase, *NA* not applicable

^*^ *p* < 0.05, ** *p* < 0.01, *** *p* < 0.001

### Multiple linear regression models of Helsinki Biobank and TSDS

To assess potential confounding factors in the Helsinki Biobank and TSDS cohorts, similar multiple linear regression models were constructed for TH reactivity, with age, sex, presence of Lewy pathology in the CNS, and MSA as independent variables (Table 2). In both cohorts, CNS Lewy pathology was significantly associated with TH reactivity (*P* < 0.001), whereas age, sex, and MSA were not. Additionally, when comparing LBD with other neurodegenerative disease pathologies (AD, MSA and PSP) in the Helsinki Biobank cohort, only LBD showed lower TH reactivity compared to the other pathology groups although the difference was not statistically significant (Supplementary Fig. 8).

**Table 2:** Multiple linear regression models of relative myocardial TH reactivity in Helsinki Biobank and TSDS cohorts, left ventricle

	Helsinki Biobank	TSDS
Constant	−1.461 [−3.626, 0.704]	−1.686*** [−2.289, −1.084]
Age at death	−0.020 [−0.049, 0.009]	−0.001 [−0.009, 0.007]
Female sex	0.463 [−0.034, 0.960]	−0.110 [−0.304, 0.084]
LP positive	−1.236*** [−1.834, −0.637]	−0.574*** [−0.758, −0.389]
MSA	−0.135 [−0.943, 0.674]	−0.316 [−0.800, 0.168]
Num.Obs	87	127

Multiple linear regression models of relative myocardial TH reactivity in the Helsinki Biobank and TSDS cohorts in left ventricle samples. Coefficient estimate and 95% confidence intervals are shown. *P* values are shown as stars. A Box–Cox transformation was performed on the dependent variable in both cohorts to alleviate heteroskedasticity. Results are shown in the transformed form as it is not possible to back-transform the coefficient estimate and confidence intervals. Due to this, the estimates between the cohorts are not directly comparable

*TH* tyrosine hydroxylase, *LP positive* CNS Lewy pathology-positive, *MSA* multiple system atrophy

^*^ *p* < 0.05, ** *p* < 0.01, *** *p* < 0.001

### Association of cardiac TH reactivity with severity of CNS Lewy pathology

**Fig. 4:**
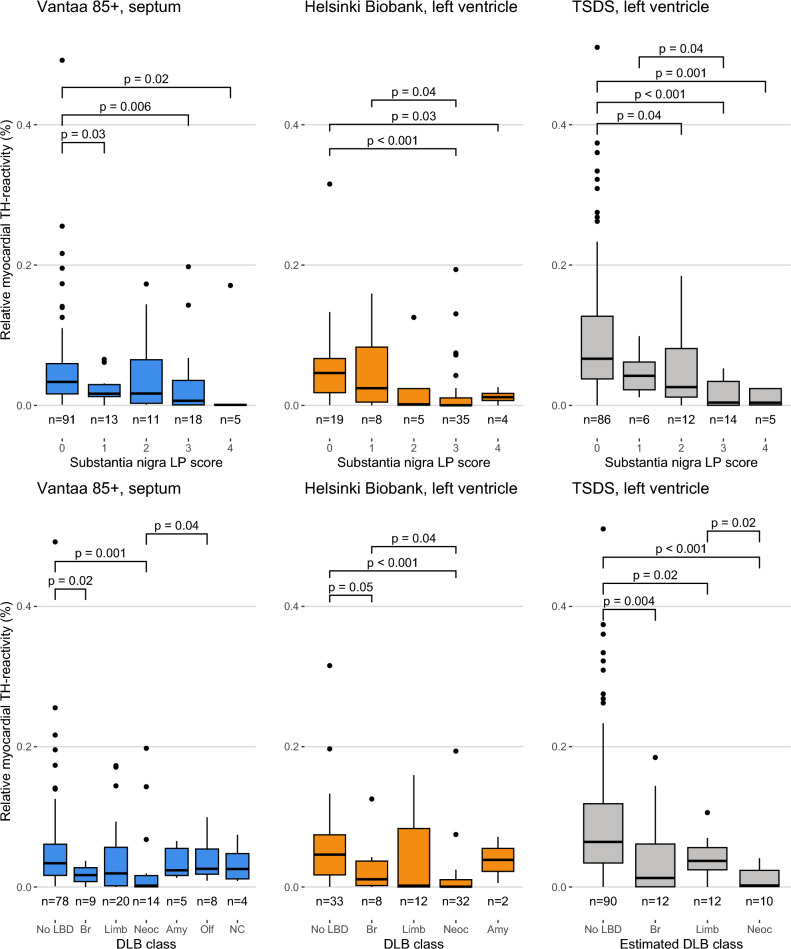
Relative myocardial TH reactivity compared to the semi-quantitative substantia nigra LP scores and DLB consortium classification. Relative myocardial TH reactivity is defined as the area of TH-positive staining divided by analyzed tissue area, excluding the tissue and TH-positive staining in larger nerve fascicles (diameter > 20 µm). Box-and-whiskers plots with median, 1st to 3rd interquartile range, and minimum and maximum. Outliers shown as dots. P values calculated using Mann–Whitney U test, only comparisons where *P* < 0.05 are shown. MSA cases are excluded in the substantia nigra LP analyses. Substantia nigra was missing for three non-MSA Helsinki Biobank cases. In TSDS, the estimated DLB consortium class is based on LP positivity in the substantia nigra, hippocampus and frontal cortex (No LBD = SN-, HC-, FC-; Br = SN + , HC-, FC-; Limb = SN + , HC + , FC-; Neoc = SN + , HC + , FC +). Cases with any of SN, HC or FC missing are excluded (*n* = 3). *SN* substantia nigra, *HC* hippocampus, *FC* frontal cortex, *DLB* dementia with Lewy bodies, *LBD* Lewy body disease, *Br* brainstem-predominant, *Limb* limbic, *Neoc* diffuse neocortical, *Amy* Amygdala-predominant, *Olf* olfactory bulb only, *NC* non-classifiable

To assess how the severity of CNS Lewy pathology is associated with myocardial TH reactivity, we next compared the TH reactivity in the heart and the semi-quantitative Lewy pathology severity score of the substantia nigra. In all three cohorts, TH reactivity showed a consistent downward trend as the severity of substantia nigra Lewy pathology increased (Fig. 4). We also assessed the association between DLB consortium classes and TH reactivity. In all cohorts, the loss of TH reactivity became more prominent as the severity of CNS Lewy pathology increased (Fig. 4). The amygdala-predominant (Vantaa 85 + and Helsinki Biobank cohorts) and olfactory bulb only cases (Vantaa 85 +) showed similar levels of TH reactivity as the no LBD group. Mean, standard deviation, median, and interquartile range of these groups are presented in Supplementary Table 9.

### Association of cardiac TH reactivity with cardiac α-syn pathology

**Fig. 5:**
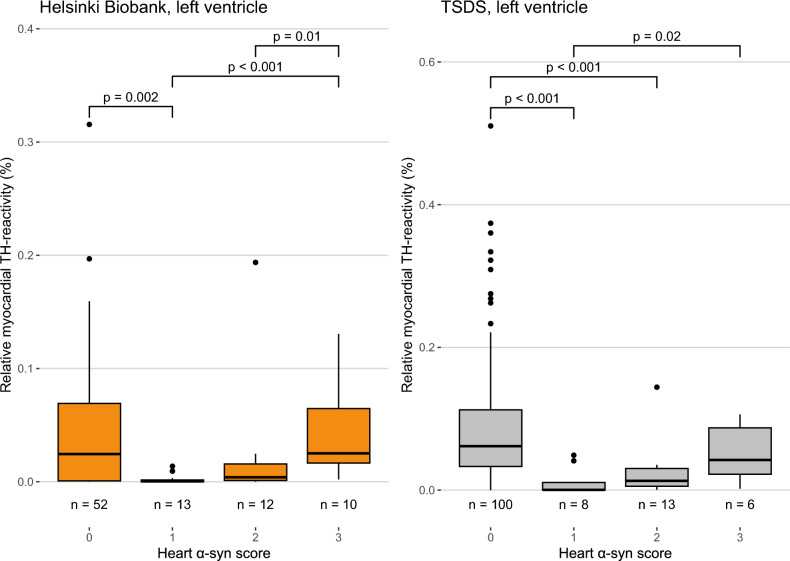
Severity of α-syn pathology in the heart compared to relative myocardial TH reactivity. Relative myocardial TH reactivity is defined as the area of TH-positive staining divided by analyzed tissue area, excluding the tissue and TH-positive staining in larger nerve fascicles (diameter > 20 µm). Box-and-whiskers plots with median, 1st to 3rd interquartile range, and minimum and maximum. Outliers shown as dots. α-syn pathology in the heart was scored on a scale of 0–3 (0 = none; 1 = focal, sparse; 2 = local, moderate; 3 = widespread, severe). Data of the septal samples of the Vantaa 85 + study are not shown due to very low number of cases with cardiac α-syn pathology. *P *values calculated using Mann–Whitney U test, only comparisons where *P* < 0.05 are shown

We next assessed cardiac α-syn positivity in all three cohorts. In the left ventricle samples of the Helsinki Biobank and TSDS samples, subjects with sparse or moderate α-syn pathology (scores 1 and 2) had the least TH reactivity, whereas subjects with abundant α-syn pathology (score 3) had higher TH reactivity (Fig. 5). This is in accordance with a previous finding showing that cardiac α-syn pathology is most abundant in early cases with relatively preserved TH reactivity, whereas cases with sparse cardiac α-syn pathology show more severe loss of TH reactivity [26]. Mean, standard deviation, median, and interquartile range of these groups are presented in Supplementary Table 10. In the septal samples of the Vantaa 85 + study, only 10 cases were positive and due to low numbers, statistical analyses were not carried out. The low number of positive cases is most likely due to the fact that the septum excludes large epicardial nerve fascicles, where most cases show α-syn pathology in the heart.

## Discussion

To our knowledge, this is the first study focused on quantitative assessments of distal sympathetic axons among the cardiomyocytes, as represented by relative TH reactivity, in the context of cardiac sympathetic denervation/dysfunction in LBD. Previous histological analyses have focused on semi-quantitative or quantitative assessments of larger epicardial sympathetic nerve fascicles, which may however, be confounded by considerable variance in their number, size, and shape in tissue sections [1, 22, 26, 32, 36]. Sympathetic axons are normally abundant within the myocardium and likely the first nervous structures to degenerate as they represent the most distal part of the nervous system. Thus, quantifying them could provide a more sensitive and reliable method to detect denervation/dysfunction. To assess this hypothesis, we developed an artificial intelligence-based image recognition algorithm that allows for quantitative analysis of myocardial TH reactivity. Supporting the reliability of the algorithm, the finding of diminished cardiac sympathetic innervation in subjects with Lewy pathology was reproduced in all three independent cohorts (Fig. 2). Taken together, our findings clarify the histopathological changes in LBD patients at the myocardial level, potentially explaining some of the cardiac manifestations seen in LBD [7, 12, 16, 35].

One of the most important findings of our study is that histopathologically assessed LBD subtypes differ in their effects on cardiac sympathetic innervation at tissue level. In the Vantaa 85 + cohort, the caudo-rostral subtype was strongly associated with diminished myocardial TH reactivity, while the amygdala-based subtype showed no such association. This finding concurs with a recent imaging study showing that subjects with isolated REM-sleep behavioral disorder, a suggested clinical marker for the body-first (caudo-rostral) subtype, show early cardiac sympathetic dysfunction [13]. Given that histological loss of TH-positivity has previously been shown to be associated with reduced MIBG uptake [36], our study provides a crucial link between the imaging literature and the caudo-rostral and amygdala-based (or brain- and body-first) subtypes of pathology observed in post-mortem cases.

In the present study, the caudo-rostral LBD subtype showed a strong independent effect in multiple linear regression models. Myocardial infarction was also statistically significantly associated with loss of TH reactivity albeit with a lower estimated effect. Senile systemic amyloidosis and diabetes medication did not show a statistically significant association. These results indicate that caudo-rostral subtype and myocardial infarctions are the most important causes for myocardial sympathetic denervation/dysfunction in the oldest-old population although further studies are needed to verify this finding in other datasets. As the caudo-rostral subtype is common in the 85-year-old and older population (frequency 27% in the Vantaa 85 + cohort), its associated cardiac manifestations may be more common than previously understood, warranting further studies.

In some countries, MIBG is a standard diagnostic tool for LBD and has been shown to have a sensitivity of 70–90% and specificity of 79–100% in distinguishing clinical PD and DLB from other neurodegenerative diseases, with a sensitivity of 70–80% and specificity of 80–96% for neuropathologically confirmed LBD [22, 38, 39, 41]. Our result that different LBD subtypes differ in myocardial TH reactivity may explain why not all LBD cases are detected using MIBG. Our results are also consistent with previous studies showing that subjects with the brain-first LBD subtype show less peripheral pathology [2, 30]. This may result in cardiac MIBG scintigraphies and seed amplification assays of skin and cerebrospinal fluid failing to detect brain-first cases reliably, which should be taken into account when interpreting the results of these procedures [20]. Going further, different subtypes may even require subtype-specific treatment protocols. In addition, we found that cases with sparse cardiac α-syn showed more severe degeneration of the TH-stained nerve fibers compared to cases with abundant cardiac α-syn pathology. Although a mechanistic link between reduced TH reactivity and cardiac α-syn has not been demonstrated, our findings are in line with previous observations, suggesting that cardiac α-syn pathology originates in paravertebral sympathetic ganglia, eventually resulting in degeneration of the sympathetic axons and removal of α-syn from the heart [26]. In line with a previous study, MSA cases did not show degeneration of TH-reactive fibers [26]. Furthermore, subjects with AD or PSP did not show evidence of diminished TH reactivity (Supplementary Fig. 8), supporting an LBD-specific effect.

Our study has several strengths and limitations. Although three independent autopsy cohorts were analyzed, samples were only available from one anatomical cardiac region (either left ventricle or septum) in each cohort. Nevertheless, the results were similar and robust in both regions studied. In the Vantaa 85 + study, the septum sample was collected in a standardized way from the same anatomical area regardless of any macroscopic change. However, in the Helsinki Biobank and TSDS cases, the left ventricle samples were often collected from areas where a macroscopic change (most commonly an infarct) was seen, and this may have affected some of the results. A major strength of the present study is that the Vantaa 85 + study and TSDS can be regarded as being population-representative in nature. In contrast, the Helsinki Biobank sample is highly selected, but allows us to compare LBD to other neurodegenerative diseases. The TSDS and Helsinki Biobank samples did not include olfactory bulb and in many cases, the amygdala. Therefore, we cannot rule out early pathology in these brain areas in LP-negative cases. In addition, limited clinical data were available for all datasets. Although information on diabetes medication was available for the Vantaa 85 + study, more comprehensive information (e.g., type and duration of diabetes) would be needed to thoroughly study its effect. In addition, a recent study suggested that the body-first LBD subtype further subdivides into sympathetic-predominant and parasympathetic-predominant patterns [2]. Both patterns are believed to originate in the periphery, and in the sympathetic-predominant pattern, isolated Lewy pathology is observed in the heart and sympathetic trunk, whereas in the parasympathetic-predominant pattern, initial Lewy pathology is found in the brainstem [2]. As sympathetic trunk samples were not available in our datasets, these newly described subtypes could not be reliably studied. However, we observed singular cases harboring α-syn pathology in the heart, but not in the CNS (Supplementary Results, Supplementary Fig. 9), potentially representing the sympathetic-predominant pattern. The question of how LBD might affect cardiac parasympathetic innervation remains unanswered and future studies are needed to fully understand the effects of LBD on the cardiac autonomic nervous system. Finally, the neural network algorithm in this study was produced using the commercial Aiforia platform, which is not freely available nor open source. Replication of these results using publicly available algorithms is warranted.

## Supplementary Information

Below is the link to the electronic supplementary material.

Supplementary file1 (DOCX 25260 KB)

## Data Availability

All data used and analyzed in this study are available from the corresponding author upon reasonable request.
