# Supplementary material for: Myocardial sympathetic distal axon loss in subjects with Lewy pathology in three autopsy cohorts
Source: Acta Neuropathol. 2025 Aug 1;150(1):11. doi: 10.1007/s00401-025-02918-y (PMC12316836; doi:10.1007/s00401-025-02918-y)
Supplement: Supplementary file 1 — Supplementary file1 (DOCX 25260 KB) [file 401_2025_2918_MOESM1_ESM.docx]

**Supplementary material**

**Myocardial sympathetic distal axon loss in subjects with Lewy pathology in three autopsy cohorts**

Ville Kivistö^1^, Benjamin Englert^1,2^, Jarno Tuimala^1^, Eloise Kok^3^, Henri Puttonen^1,2^, Anna Raunio^1,2^, Pekka J. Karhunen^3,4,5^, Maria K. Lehtinen^6^, Per Borghammer^7^, Ella Ahvenainen^1^, Kia Colangelo^1^, Sara Savola^1,2^, Maarit Tanskanen^1^, Karri Kaivola^8^, Pentti J. Tienari^9^, Darshan Kumar^10^, Anders Paetau^1^, Olli Tynninen^1,2^, Mikko I. Mäyränpää^1,2^, Tuomo Polvikoski^11^, Liisa Myllykangas^1,2^

1. Department of Pathology, University of Helsinki, 00014 Helsinki, Finland
2. HUS Diagnostic Center at Helsinki University Hospital, 00029 Helsinki, Finland
3. Faculty of Medicine and Health Technology, Tampere University, 33100 Tampere, Finland
4. Finnish Cardiovascular Research Center – 33014 Tampere, Finland
5. Fimlab Laboratories Ltd., Wellbeing Services County of Pirkanmaa, 33520 Tampere, Finland
6. Department of Pathology, Boston Children’s Hospital and Harvard Medical School, Boston, MA 02115, USA
7. Institute of Clinical Medicine, Aarhus University, 8200, Aarhus, Denmark
8. Translational Immunology, Research Programs Unit, University of Helsinki, 00014 Helsinki, Finland
9. Brain Center, Neurology, HUS, Helsinki University Hospital, 00014 Helsinki, Finland
10. Aiforia Technologies Oyj., 00150 Helsinki, Finland
11. Newcastle University Translational and Clinical Research Institute, University of Newcastle, Newcastle upon Tyne NE1 7RU, United Kingdom

**Corresponding author:** Liisa Myllykangas, Department of Pathology, University of Helsinki and HUS Diagnostic Center, Helsinki University Hospital. POB 21, 00014 Helsinki, Finland. Tel. +358-504482805, Email liisa.myllykangas@helsinki.fi

**Supplementary methods**

## **Image recognition algorithm training and validation**

**We developed an image recognition algorithm based on a neural network tool (Aiforia**®)**. All cardiac slides were digitalised with a scanner (**3D HISTECH Pannoramic 250 Flash III). **The algorithm consists of three nested layers with one or two classes in each layer. The first layer contains the class “Tissue”, which roughly separates tissue from background. The second layer contains the classes “Nerve fascicles” (fascicles with diameter** > 20 µm) **and “Non-fascicle tissue”, allowing the algorithm to distinguish TH-reactivity in larger nerve fascicles from the smaller distal axons. The third layer has one class that recognises the TH-positive staining, shared by the second layer classes.**

**The training set consisted of 33 whole slide images (WSI) with 9 WSIs from Vantaa 85+ study, 20 from Helsinki Biobank and 4 from TSDS. In the final training, the algorithm was trained for 2715 iterations over 1334 training regions. The area of training regions was 145.88 mm^2^ for the first layer, 55.38 mm^2^ for the second layer, and 0.73 mm^2^ for the third layer.**

Validation of the algorithm was performed by comparing the results of the algorithm to annotations made by board-certified pathologists (LM, MM, HP). The pathologists did not receive any clinical or pathological information about the cases. The validation set included 10 slides from each of the three cohorts not used in training for a total of 30 validation slides. The results of the algorithm in the validation areas were compared to annotations made by the pathologists. From the validation data, we computed parameter values for each layer and class separately (Supplementary Table 6).

In the parameters, total layer area error refers to the sum of all false positive (FP) and false negative (FN) areas in that layer (including all classes), divided by the total validation area in that layer. Class specific error refers to the sum of the FP area and the FN area of the respective class divided by all the area annotated by the pathologist as the class in question. Class FP% and class FN% are calculated as class FP or FN area, divided by the class area annotated by the pathologist. Precision is calculated as area correctly recognised by the algorithm, divided by all area recognised by the algorithm. Sensitivity is calculated by dividing correctly recognised class area, by the sum of correctly recognised class area and area that was falsely not recognised.

**Validation parameters confirmed that the “Non-fascicle tissue” class was performing reliably, with an average sensitivity of 99.21% and average precision of 98.23% indicating excellent performance (Supplementary Table 6). The “Nerve fascicles” class also performed well with an average sensitivity of 91.32% and an average precision of 91.15%. The “TH-positive” class showed good average sensitivity and precision at 78.90% and 83.96%. The “Tissue” class displayed excellent performance and parameters with average sensitivity and precision of 99.83% and 99.25%. Visual inspection of analysis areas from samples not in the training set confirmed that the classes were being recognised correctly (See Supplementary Figs. 6 and 7).**

**Supplementary results**

# **Helsinki Biobank Cohort: Myocardial TH-reactivity in subjects with pure LBD, MSA, AD and PSP**

We wanted to see how the different neurodegenerative diseases (LBD, MSA, AD, PSP) affected myocardial TH-reactivity in the Helsinki Biobank cohort without concurrent pathologies. For this analysis, we included cases with only one type of pathology present. In our analyses, LBD stood out as clearly showing lower TH-reactivity, while the other diagnosis groups (MSA, PSP, or AD) did not seem to differ from each other or the cases without any neurodegenerative diseases (Supplementary Fig. 8), although the differences between the groups were not statistically significant, possibly due to low numbers.

**Isolated cardiac α-syn pathology**

Previous studies have reported cases with isolated cardiac α-syn pathology (without CNS pathology) [1, 7]. In our cohorts such cases were rare. However, we found one case with isolated cardiac α-syn in the Vantaa 85+ cohort (Supplementary Fig. 9). In addition, one case in the Helsinki Biobank cohort had α-syn pathology in the heart, but only minimal Lewy pathology in the brain (a couple of neurites/Lewy bodies in medulla). There were also four cases with α-syn pathology only in the heart in the TSDS cohort, but it is noteworthy that TSDS subjects have not been analysed for Lewy pathology in the medulla or olfactory bulb (samples from these regions have not been collected), so we cannot exclude that these cases might have had Lewy pathology in either of these regions.

**Supplementary Table 1. Demographic details of the Vantaa 85+ cohort.**

|  | | LP negative | | | LP positive | | |
| --- | --- | --- | --- | --- | --- | --- | --- |
|  |  | Mean | n (%) |  | Mean | n (%) |  |
| Age at death | | 93.4 |  |  | 93.8 |  |  |
| Sex | Male |  | 7 | (9.0%) |  | 14 | (23.3%) |
|  | Female |  | 71 | (91.0%) |  | 46 | (76.7%) |
|  | Total |  | 78 |  |  | 60 |  |
| Myocardial infarction | No |  | 47 | (60.3%) |  | 37 | (61.7%) |
|  | Yes |  | 28 | (35.9%) |  | 17 | (28.3%) |
|  | Data missing |  | 3 | (3.8%) |  | 6 | (10.0%) |
|  | Total |  | 78 |  |  | 60 |  |
| Diabetes medication | No |  | 61 | (78.2%) |  | 48 | (80.0%) |
|  | Yes |  | 17 | (21.8%) |  | 12 | (20.0%) |
|  | Total |  | 78 |  |  | 60 |  |
| Senile systemic amyloidosis | No |  | 51 | (65.4%) |  | 45 | (75.0%) |
|  | Yes |  | 22 | (28.2%) |  | 11 | (18.3%) |
|  | Data missing |  | 5 | (6.4%) |  | 4 | (6.7%) |
|  | Total |  | 78 |  |  | 60 |  |
| DLB consortium class | No LBD |  | 78 | (100.0%) |  | 0 | (0.0%) |
|  | Brainstem-predominant |  | 0 | (0.0%) |  | 9 | (15.0%) |
|  | Limbic |  | 0 | (0.0%) |  | 20 | (33.3%) |
|  | Diffuse neocortical |  | 0 | (0.0%) |  | 14 | (23.3%) |
|  | Amygdala-predominant |  | 0 | (0.0%) |  | 5 | (8.3%) |
|  | Olfactory bulb only |  | 0 | (0.0%) |  | 8 | (13.3%) |
|  | Non-classifiable |  | 0 | (0.0%) |  | 4 | (6.7%) |
|  | Total |  | 78 |  |  | 60 |  |
| LBD subtype | No LP |  | 78 | (100.0%) |  | 0 | (0.0%) |
|  | Caudo-rostral |  | 0 | (0.0%) |  | 36 | (60.0%) |
|  | Amygdala-based |  | 0 | (0.0%) |  | 24 | (40.0%) |
|  | Total |  | 78 |  |  | 60 |  |

Demographic details of the Vantaa 85+ cohort. Missing data indicated where applicable. Abbreviations: DLB = dementia with Lewy bodies, LBD = Lewy body disease, LP = Lewy pathology, as assessed in the central nervous system.

**Supplementary Table 2. Demographic details of the Helsinki Biobank cohort**

|  | | LP negative | | | LP positive | | |
| --- | --- | --- | --- | --- | --- | --- | --- |
|  |  | Mean | n (%) |  | Mean | n (%) |  |
| Age at death | | 71.3 |  |  | 78.1 |  |  |
| Sex | Male |  | 12 | (36.4%) |  | 31 | (57.4%) |
|  | Female |  | 21 | (63.6%) |  | 23 | (42.6%) |
|  | Total |  | 33 |  |  | 54 |  |

Demographic details of the Helsinki Biobank cohort. LP = Lewy pathology, as assessed in the central nervous system.

**Supplementary Table 3. Neuropathological diagnoses of the Helsinki Biobank cohort**

|  | | Sex | | |  | Age at death |
| --- | --- | --- | --- | --- | --- | --- |
|  |  | Male | Female | Total |  | Mean |
|  |  | n | n | n (%) |  |  |
| DLB consortium class | Brainstem-predominant | 2 | 6 | 8 | (9.2%) | 80.7 |
|  | Limbic | 7 | 5 | 12 | (13.8%) | 80.2 |
|  | Diffuse neocortical | 22 | 10 | 32 | (36.8%) | 76.8 |
|  | Amygdala-predominant | 0 | 2 | 2 | (2.3%) | 75.1 |
| MSA |  | 4 | 9 | 13 | (14.9%) | 67.4 |
| PSP |  | 5 | 4 | 9 | (10.3%) | 73.4 |
| AD |  | 2 | 5 | 7 | (8.1%) | 74.2 |
| No neurodegenerative disease | | 1 | 3 | 4 | (4.6%) | 74.3 |
| Total | | 43 | 44 | 87 |  | 75.5 |

Neuropathological details of the Helsinki Biobank cohort. DLB consortium class shown for the Lewy body disease cases. DLB = dementia with Lewy bodies, MSA = multiple system atrophy, PSP = progressive supranuclear palsy, AD = Alzheimer's disease.

**Supplementary Table 4. Demographic details of the TSDS cohort**

|  | | LP negative | | | LP positive | | | MSA |  |  |
| --- | --- | --- | --- | --- | --- | --- | --- | --- | --- | --- |
|  |  | Mean | n (%) |  | Mean | n (%) |  | Mean | n (%) |  |
| Age at death | | 74.0 |  |  | 75.7 |  |  | 64.8 |  |  |
| Sex | Male |  | 62 | (72.2%) |  | 27 | (73.0%) |  | 3 | (75.0%) |
|  | Female |  | 24 | (27.8%) |  | 10 | (27.0%) |  | 1 | (25.0%) |
|  | Total |  | 86 |  |  | 37 |  |  | 4 |  |

Demographic details of the TSDS cohort. LP = Lewy pathology, as assessed in the central nervous system, MSA = multiple system atrophy, TSDS = Tampere Sudden Death Study.

**Supplementary Table 5. Descriptions of the variables used in the analyses**

| **Vantaa 85+** | |  |
| --- | --- | --- |
|  | Age at death | Age at death, calculated from day of birth and day of death |
|  | Sex | Sex of the subject |
|  | LP positive | Presence of Lewy pathology in immunohistochemical staining in any of the central nervous system samples, including: olfactory bulb/peduncle, cortex (frontal, temporal, parietal, cingulate), entorhinal cortex, CA2 area of hippocampus, amygdala, substantia nigra, spinal cord |
|  | LP negative | No presence of Lewy pathology in the aforementioned stainings |
|  | DLB consortium classification | Classified according to the guidelines published in the DLB consortium consensus reports[3–5] |
|  | LBD subtype | As assessed by Raunio et al. [6] with slight modifications, see section “Classification of LBD subtypes in the Vantaa 85+ cohort” in the main text. |
|  | Substantia nigra LP score | Presence of Lewy pathology in the substantia nigra, scored from 0 to 4 according to DLB classification guidelines[4] |
|  | Cardiac α-syn pathology score | Scored from 0 to 3, where 0 = no pathology, 1 = focal, sparse pathology, 2 = local, moderate pathology, 3 = widespread, severe pathology |
|  | Myocardial infarction | Assessed macroscopically at autopsy and confirmed histologically[8] |
|  | Diabetes medication | Use of blood glucose-lowering medication or insulin[8] |
|  | Senile systemic amyloidosis | Assessed by detection of amyloid in Congo red staining, and confirmed as senile systemic amyloidosis by transthyretin immunohistochemistry[8] |
|  |  |  |
| **Helsinki Biobank** | |  |
|  | Age at death | Age at death, calculated from day of birth and day of death |
|  | Sex | Sex of the subject |
|  | LP positive | Diagnosed as LBD at autopsy by a neuropathologist (based on autopsy report) |
|  | LP negative | No LBD diagnosis at autopsy by a neuropathologist and negative brainstem (substantia nigra, or pons and medulla if substantia nigra was not available, n = 2) alpha-synuclein immunohistochemistry |
|  | MSA | Diagnosed as multiple system atrophy at autopsy, co-occurring Lewy pathology accepted in brainstem alpha-synuclein immunohistochemistry |
|  | Substantia nigra LP score | Presence of Lewy pathology in the substantia nigra, scored from 0 to 4 according to DLB classification guidelines[4] |
|  | Cardiac α-syn pathology score | Scored from 0 to 3, where 0 = no pathology, 1 = focal, sparse pathology, 2 = local, moderate pathology, 3 = widespread, severe pathology |
|  | DLB consortium classification | Defined by presence of Lewy pathology at brainstem, limbic or frontal cortex, as reported by a neuropathologist at the autopsy report[3–5] |
|  |  |  |
| **TSDS** |  |  |
|  | Age at death | Age at death, calculated from day of birth and day of death, rounded to the nearest whole number |
|  | Sex | Sex of the subject |
|  | LP positive | Based on presence of Lewy pathology in brainstem tissue sections alongside the presence of neuromelanin positive stained cells in α-syn immunohistochemical staining[2] |
|  | LP negative | No Lewy pathology in the aforementioned staining |
|  | MSA | Presence of glial cytoplasmic inclusions in α-syn immunohistochemical staining[2] |
|  | Substantia nigra LP score | Presence of Lewy pathology in the substantia nigra, scored from 0 to 4 according to DLB classification guidelines[4] |
|  | Cardiac α-syn pathology score | Scored from 0 to 3, where 0 = no pathology, 1 = focal, sparse pathology, 2 = local, moderate pathology, 3 = widespread, severe pathology |
|  | Estimated DLB classification | Based on presence of Lewy pathology in immunohistochemical staining in the substantia nigra, hippocampus and frontal cortex[2] |

**Supplementary Table 6. Validation parameters of the Aiforia image recognition algorithm**

|  |  |  | **Pathologist** | |  |
| --- | --- | --- | --- | --- | --- |
| **Class** | **Parameter** | **1** | **2** | **3** | **Average** |
| **TH-positive** |  |  |  |  |  |
|  | Total layer error % | 0.28 % | 0.30 % | 0.29 % | 0.29 % |
|  | Class specific error % | 40.04 % | 35.25 % | 34.93 % | 36.74 % |
|  | Class FP % | 23.96 % | 11.18 % | 11.66 % | 15.60 % |
|  | Class FN% | 16.08 % | 24.07 % | 23.27 % | 21.14 % |
|  | Precision | 77.84 % | 87.19 % | 86.84 % | 83.96 % |
|  | Sensitivity | 83.96 % | 75.98 % | 76.78 % | 78.90 % |
| **Non-fascicle tissue** |  |  |  |  |  |
|  | Total layer error % | 3.06 % | 3.16 % | 2.80 % | 3.01 % |
|  | Class specific error % | 2.60 % | 2.80 % | 2.34 % | 2.58 % |
|  | Class FP % | 1.82 % | 2.16 % | 1.38 % | 1.79 % |
|  | Class FN% | 0.78 % | 0.64 % | 0.96 % | 0.79 % |
|  | Precision | 98.20 % | 97.87 % | 98.62 % | 98.23 % |
|  | Sensitivity | 99.22 % | 99.36 % | 99.04 % | 99.21 % |
| **Nerve fascicles** |  |  |  |  |  |
|  | Total layer error % | 3.06 % | 3.16 % | 2.80 % | 3.01 % |
|  | Class specific error % | 19.35 % | 16.14 % | 17.26 % | 17.58 % |
|  | Class FP % | 10.75 % | 7.21 % | 8.73 % | 8.90 % |
|  | Class FN% | 8.60 % | 8.92 % | 8.53 % | 8.68 % |
|  | Precision | 89.50 % | 92.67 % | 91.27 % | 91.15 % |
|  | Sensitivity | 91.42 % | 91.08 % | 91.46 % | 91.32 % |
| **Tissue** |  |  |  |  |  |
|  | Total layer error % | 0.59 % | 0.62 % | 0.56 % | 0.59 % |
|  | Class specific error % | 0.92 % | 0.98 % | 0.88 % | 0.93 % |
|  | Class FP % | 0.77 % | 0.73 % | 0.76 % | 0.75 % |
|  | Class FN% | 0.15 % | 0.24 % | 0.13 % | 0.17 % |
|  | Precision | 99.23 % | 99.27 % | 99.25 % | 99.25 % |
|  | Sensitivity | 99.85 % | 99.76 % | 99.87 % | 99.83 % |

| **Supplementary Table 7. Mean, standard deviation, median, and interquartile range of TH-reactivity compared between LP positive, LP negative, and MSA groups.** | | | | | | |
| --- | --- | --- | --- | --- | --- | --- |
|  |  |  |  |  |  |  |
|  | **Vantaa 85+** |  | **Helsinki Biobank** | | **TSDS** |  |
|  | Mean [SD] | Median (IQR) | Mean [SD] | Median (IQR) | Mean [SD] | Median (IQR) |
| LP negative | 0.055 [0.071] | 0.034 (0.044) | 0.057 [0.069] | 0.038 (0.048) | 0.103 [0.098] | 0.067 (0.089) |
| LP positive | 0.034 [0.047] | 0.017 (0.039) | 0.023 [0.044] | 0.002 (0.020) | 0.034 [0.043] | 0.018 (0.044) |
| MSA | NA | NA | 0.060 [0.055] | 0.051 (0.072) | 0.043 [0.028] | 0.045 (0.038) |

**TH = tyrosine hydroxylase, LP = central nervous system Lewy pathology, MSA = multiple system atrophy, SD = standard deviation, IQR = interquartile range, NA = not applicable.**

**Supplementary Table 8. Mean, standard deviation, median, and interquartile range of TH-reactivity across the LBD subtypes in the Vantaa 85+ cohort.**

|  |  |  |
| --- | --- | --- |
| Subtype | Mean [SD] | Median (IQR) |
| No LBD | 0.055 [0.071] | 0.034 (0.044) |
| Caudo-rostral | 0.019 [0.032] | 0.003 (0.021) |
| Amygdala-based | 0.058 [0.057] | 0.029 (0.046) |

**TH = tyrosine hydroxylase, LBD = Lewy body disease, SD = standard deviation, IQR = interquartile range.**

| **Supplementary Table 9. Mean, standard deviation, median, and interquartile range of TH-reactivity according to substantia nigra LP scores and DLB consortium classification.** | | | | | | | |
| --- | --- | --- | --- | --- | --- | --- | --- |
|  |  | **Vantaa 85+** |  | **Helsinki Biobank** | | **TSDS^a^** |  |
|  |  | Mean [SD] | Median (IQR) | Mean [SD] | Median (IQR) | Mean [SD] | Median (IQR) |
| Substantia nigra semiquantitative LP score | 0 | 0.052 [0.067] | 0.034 (0.043) | 0.059 [0.070] | 0.046 (0.049) | 0.103 [0.098] | 0.067 (0.089) |
|  | 1 | 0.023 [0.020] | 0.017 (0.017) | 0.051 [0.060] | 0.025 (0.078) | 0.046 [0.032] | 0.042 (0.040) |
|  | 2 | 0.046 [0.062] | 0.017 (0.062) | 0.030 [0.054] | 0.002 (0.024) | 0.055 [0.061] | 0.026 (0.069) |
|  | 3 | 0.033 [0.055] | 0.007 (0.035) | 0.018 [0.041] | 0.000 (0.011) | 0.017 [0.020] | 0.004 (0.034) |
|  | 4 | 0.035 [0.076] | 0.001 (0.001) | 0.012 [0.011] | 0.012 (0.010) | 0.011 [0.013] | 0.004 (0.023) |
|  |  |  |  |  |  |  |  |
| DLB consortium classification | No LBD | 0.055 [0.071] | 0.034 (0.044) | 0.058 [0.063] | 0.046 (0.057) | 0.100 [0.097] | 0.064 (0.084) |
|  | Brainstem-predominant | 0.017 [0.013] | 0.017 (0.020) | 0.029 [0.042] | 0.011 (0.035) | 0.044 [0.063] | 0.013 (0.061) |
|  | Limbic | 0.042 [0.058] | 0.019 (0.055) | 0.042 [0.061] | 0.002 (0.083) | 0.042 [0.029] | 0.037 (0.032) |
|  | Diffuse neocortical | 0.032 [0.062] | 0.002 (0.016) | 0.013 [0.036] | 0.001 (0.010) | 0.012 [0.017] | 0.002 (0.023) |
|  | Amygdala-predominant | 0.035 [0.024] | 0.024 (0.039) | 0.039 [0.046] | 0.039 (0.033) | NA | NA |
|  | Olfactory bulb only | 0.039 [0.031] | 0.026 (0.036) | NA | NA | NA | NA |
|  | Non-classifiable | 0.034 [0.030] | 0.026 (0.036) | NA | NA | NA | NA |

^a^For TSDS subjects, DLB consortium classification was estimated from LP positivity in substantia nigra, hippocampus and frontal cortex. DLB = dementia with Lewy bodies, LP = Lewy pathology, TH = tyrosine hydroxylase, SD = standard deviation, IQR = interquartile range, LBD = Lewy body disease, NA = not applicable.

| **Supplementary Table 10. Semiquantitative cardiac LP score and mean, standard deviation, median, and interquartile range of TH-reactivity among Helsinki Biobank and TSDS subjects.** | | | | | |
| --- | --- | --- | --- | --- | --- |
|  |  | **Helsinki Biobank** |  | **TSDS** |  |
|  |  | Mean [SD] | Median (IQR) | Mean [SD] | Median (IQR) |
| Semiquantitative cardiac LP score | 0 | 0.047 [0.061] | 0.024 (0.068) | 0.095 [0.095] | 0.062 (0.079) |
|  | 1 | 0.002 [0.004] | 0.000 (0.001) | 0.011 [0.021] | 0.000 (0.011) |
|  | 2 | 0.023 [0.054] | 0.004 (0.015) | 0.025 [0.038] | 0.013 (0.025) |
|  | 3 | 0.042 [0.039] | 0.025 (0.048) | 0.052 [0.043] | 0.042 (0.065) |

LP = Lewy pathology, TH = tyrosine hydroxylase, SD = standard deviation, IQR = interquartile range.

**Supplementary Figure 1. Flowchart of the inclusion of Vantaa 85+ subjects in the present study.**


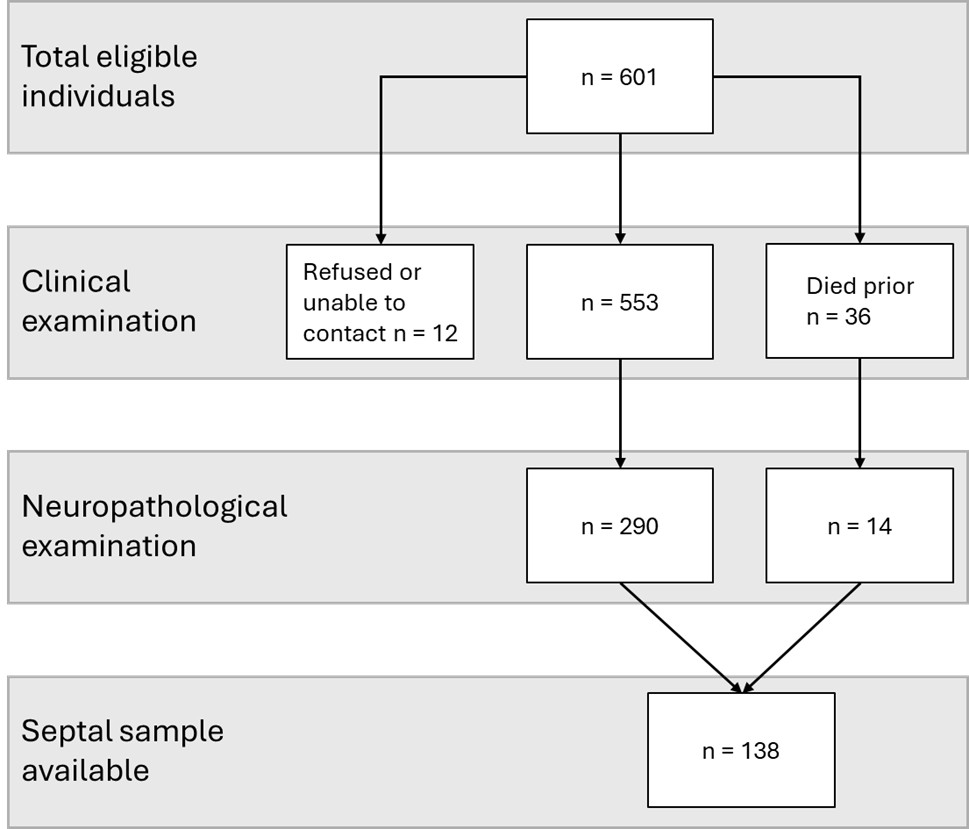


**Supplementary Figure 2. Example Lewy pathology scoring of a caudo-rostral subject from the Vantaa 85+ cohort.**

Subject with a caudo-rostral progression pattern, with a decreasing gradient of pathology from the brainstem areas towards the limbic areas and the neocortex. spin S = sacral spinal cord, spin Th = thoracic spinal cord, sn = substantia nigra, amy = amygdala, ent = entorhinal cortex, cing = cingulate cortex, temp = temporal cortex, front = frontal cortex, pariet = parietal cortex.

**Supplementary Figure 3. Example Lewy pathology scoring of an amygdala-based subject from the Vantaa 85+ cohort.**

Subject with an amygdala-based progression pattern, with a peak of pathology in the limbic areas and decreasing gradient towards the neighbouring areas. Spin S = sacral spinal cord, spin Th = thoracic spinal cord, sn = substantia nigra, amy = amygdala, ent = entorhinal cortex, cing = cingulate cortex, temp = temporal cortex, front = frontal cortex, pariet = parietal cortex.

**Supplementary Figure 4. Co-localisation of TH-positive staining with PGP9.5-positive staining**


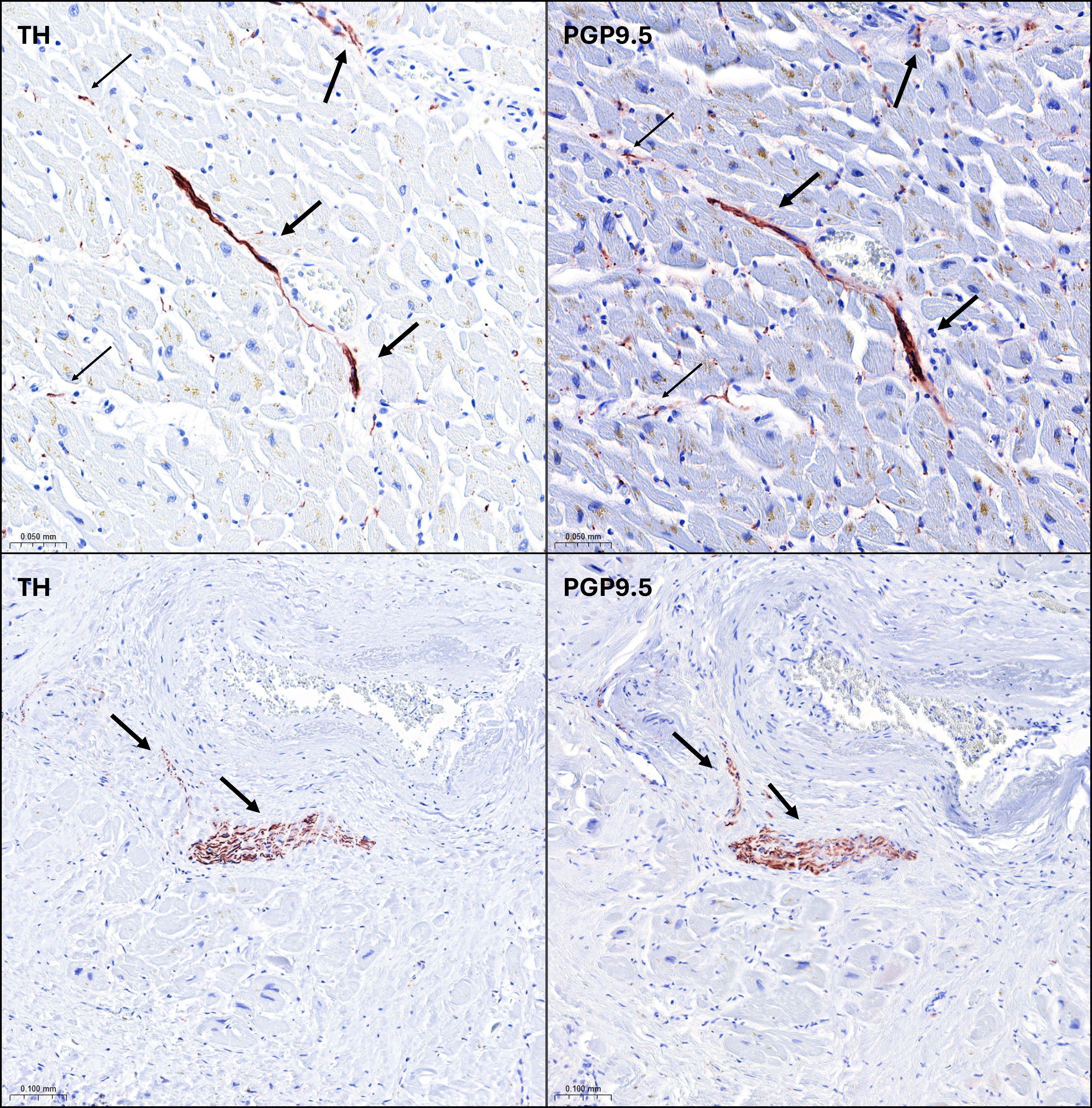


Example of co-localised TH- and PGP9.5-positive staining shown with black arrows. PGP9.5 is a neuronal marker. TH = tyrosine hydroxylase. Upper images taken at 200x magnification, lower images taken at 100x magnification.

**Supplementary Figure 5. Co-localisation of TH-positive staining with SMI-31-positive staining**


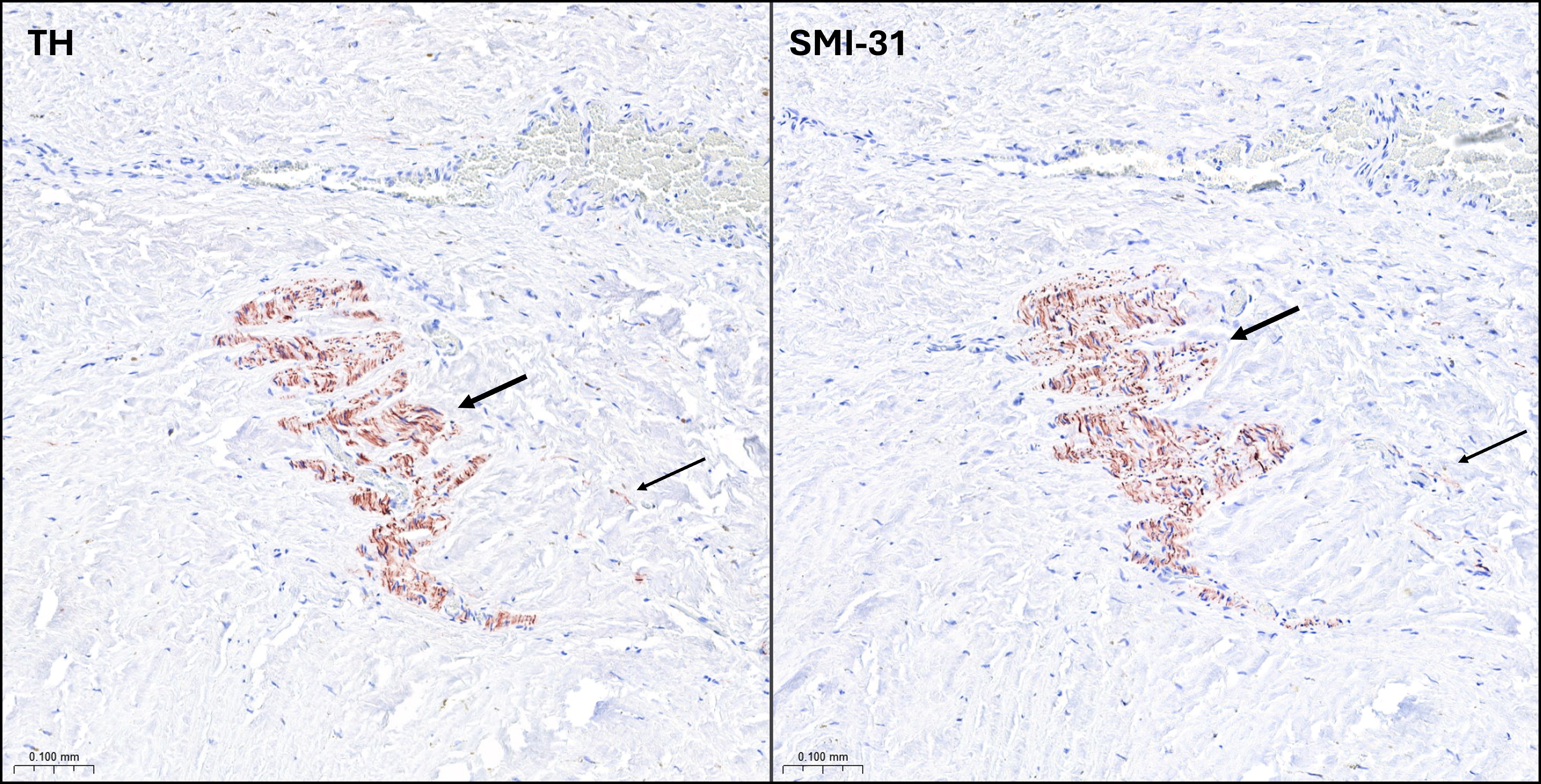


Example of co-localised TH- and SMI-31-positive staining in a large nerve shown with black arrow. SMI-31 reacts with neurofilament, seen in axons. TH = tyrosine hydroxylase. Images taken at 100x magnification.

**Supplementary Figure 6. Example performance of the TH-positive class.**


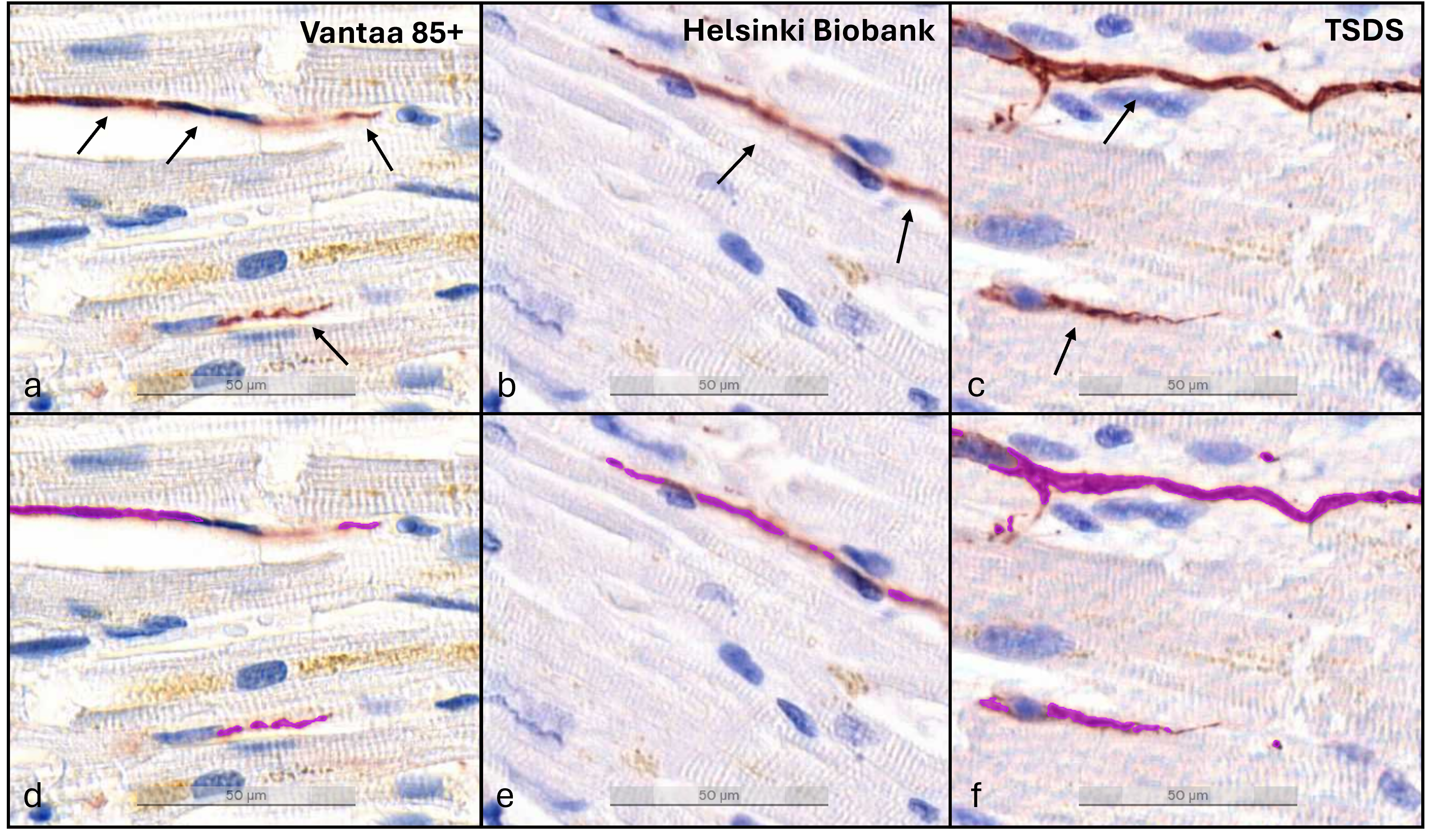


Examples of TH-positive staining shown with black arrows in each dataset. a, b, c: images showing example tissue. d, e, f: area recognised by the algorithm as TH-positive staining shown with a purple overlay. Pictures taken at 400x magnification. Scale bar shown at the bottom of each image. Images a and d demonstrate the algorithm ignoring lipofuscin, which was commonly seen in the samples of the very elderly Vantaa 85+ subjects. TH = tyrosine hydroxylase.

**Supplementary Figure 7. Example performance of the non-fascicle tissue class.**


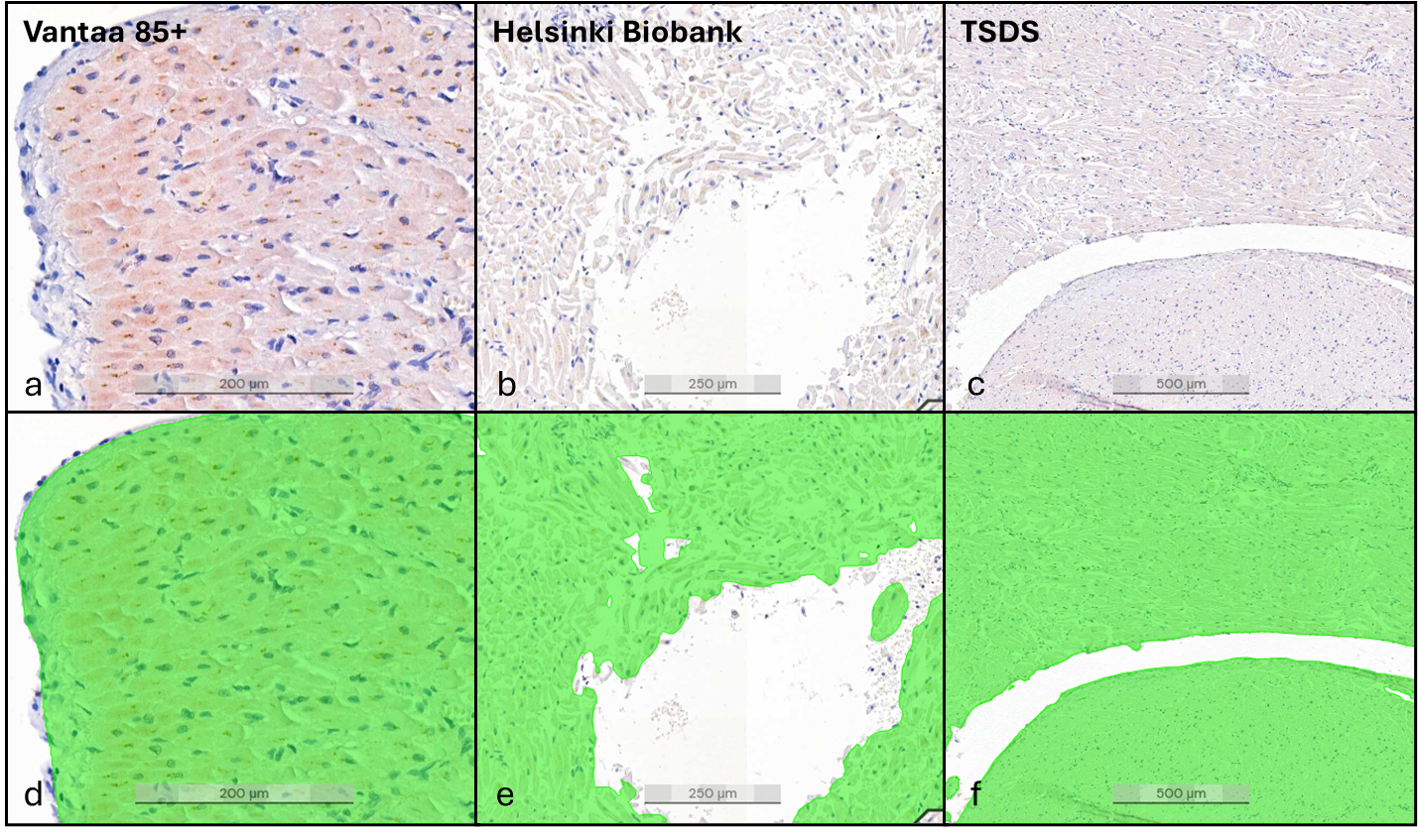


Example pictures of non-fascicle tissue from each dataset taken at varying magnifications (100x, 50x, 25x for Vantaa 85+, Helsinki Biobank and TSDS respectively). a, b, c: images from each cohort showing example tissue. d, e, f: area recognised by the algorithm as non-fascicle tissue shown with a green overlay. Scale bar is shown at the bottom for each image.

**Supplementary Figure 8. Comparison between cases without overlapping pathologies and cases without neurodegenerative diseases in the Helsinki Biobank cohort.**


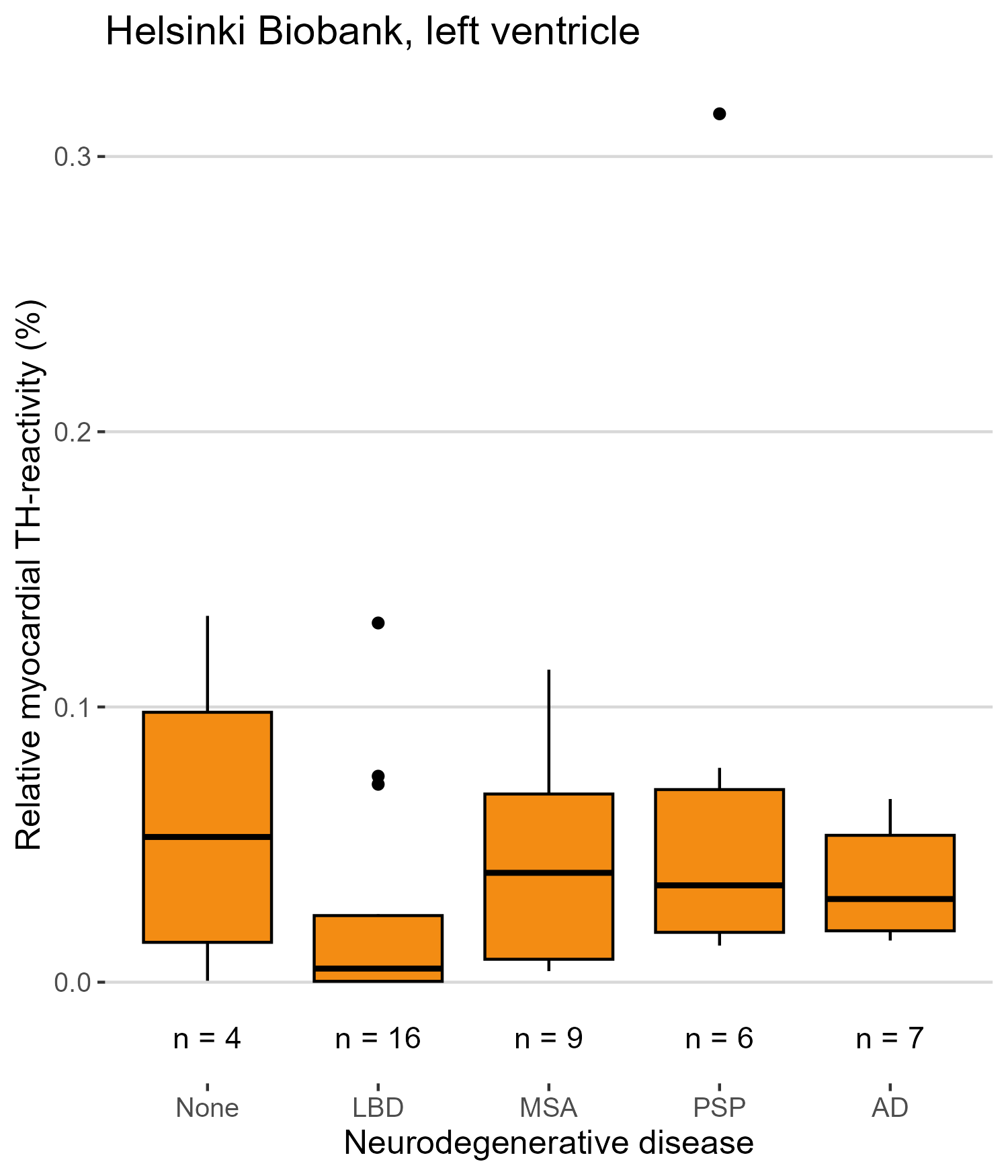


TH = tyrosine hydroxylase, LBD = Lewy body disease, MSA = multiple system atrophy, PSP = progressive supranuclear palsy, AD = Alzheimer’s disease. Group differences tested with Mann-Whitney U-test. None of the group differences were statistically significant.

**Supplementary Figure 9. Cardiac α-syn in a subject of the Vantaa 85+ study with no Lewy pathology in the CNS.**


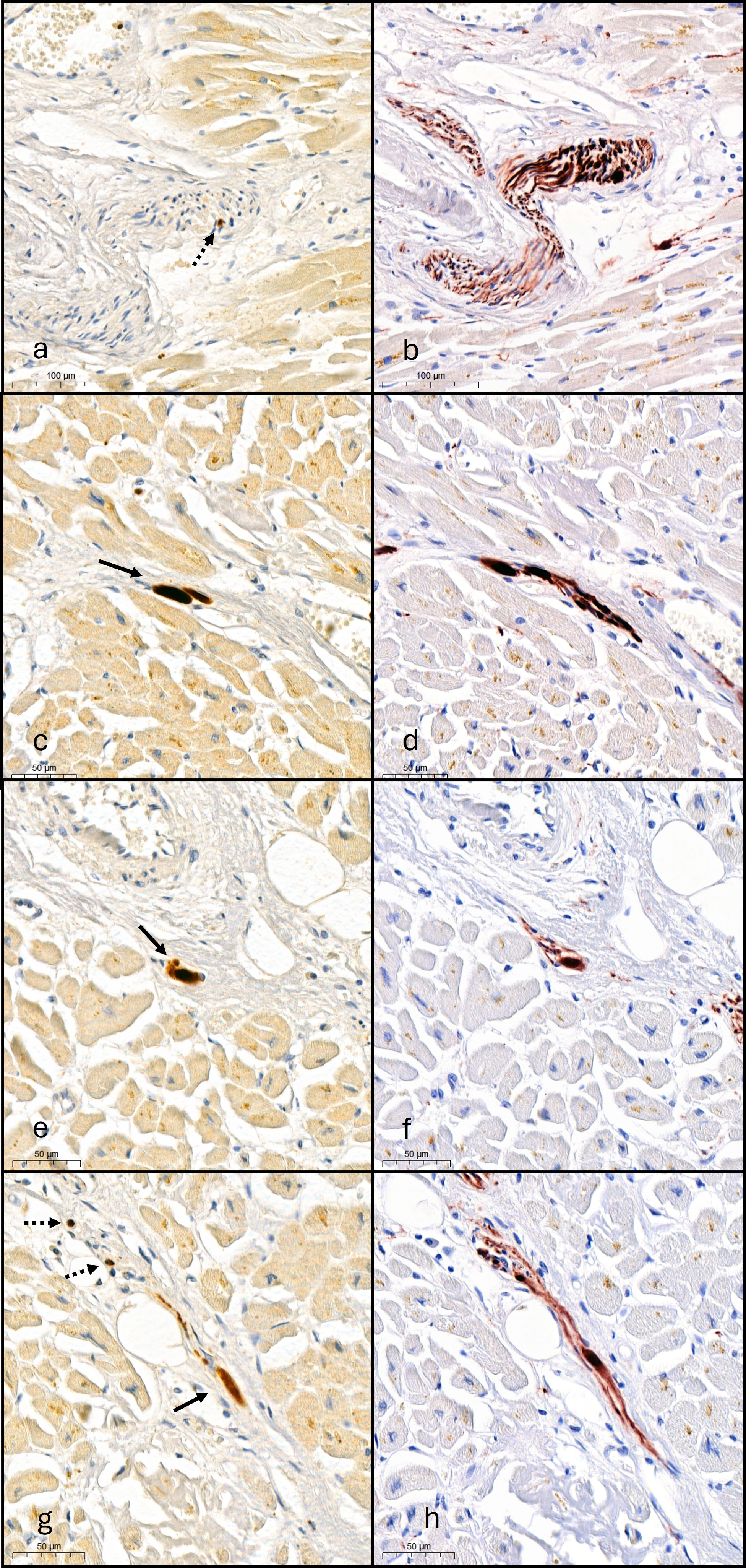


a, c, e, g: anti-α-syn staining in the myocardium showing neuritic (solid arrows) and dot-like (dashed arrows) α-syn pathology. b, d, f, h: corresponding areas stained with anti-TH-antibody shown

**References**

1. Andersen KB, Krishnamurthy A, Just MK, Van Den Berge N, Skjærbæk C, Horsager J, Knudsen K, Vogel JW, Toledo JB, Attems J, Polvikoski T, Saito Y, Murayama S, Borghammer P (2025) Sympathetic and parasympathetic subtypes of body-first Lewy body disease observed in postmortem tissue from prediagnostic individuals. Nat Neurosci. doi: 10.1038/S41593-025-01910-9

2. Kok EH, Paetau A, Martiskainen M, Lyytikäinen LP, Lehtimäki T, Karhunen P, Myllykangas L (2024) Accumulation of Lewy-Related Pathology Starts in Middle Age: The Tampere Sudden Death Study. Ann Neurol 95:843–848. doi: 10.1002/ANA.26912

3. McKeith IG, Boeve BF, Dickson DW, Halliday G, Taylor JP, Weintraub D, Aarsland D, Galvin J, Attems J, Ballard CG, Bayston A, Beach TG, Blanc F, Bohnen N, Bonanni L, Bras J, Brundin P, Burn D, Chen-Plotkin A, Duda JE, El-Agnaf O, Feldman H, Ferman TJ, Ffytche D, Fujishiro H, Galasko D, Goldman JG, Gomperts SN, Graff-Radford NR, Honig LS, Iranzo A, Kantarci K, Kaufer D, Kukull W, Lee VMY, Leverenz JB, Lewis S, Lippa C, Lunde A, Masellis M, Masliah E, McLean P, Mollenhauer B, Montine TJ, Moreno E, Mori E, Murray M, O’Brien JT, Orimo S, Postuma RB, Ramaswamy S, Ross OA, Salmon DP, Singleton A, Taylor A, Thomas A, Tiraboschi P, Toledo JB, Trojanowski JQ, Tsuang D, Walker Z, Yamada M, Kosaka K (2017) Diagnosis and management of dementia with Lewy bodies: Fourth consensus report of the DLB Consortium. Neurology 89:88–100. doi: 10.1212/WNL.0000000000004058 [doi]

4. McKeith IG, Dickson DW, Lowe J, Emre M, O’Brien JT, Feldman H, Cummings J, Duda JE, Lippa C, Perry EK, Aarsland D, Arai H, Ballard CG, Boeve B, Burn DJ, Costa D, Del Ser T, Dubois B, Galasko D, Gauthier S, Goetz CG, Gomez-Tortosa E, Halliday G, Hansen LA, Hardy J, Iwatsubo T, Kalaria RN, Kaufer D, Kenny RA, Korczyn A, Kosaka K, Lee VMY, Lees A, Litvan I, Londos E, Lopez OL, Minoshima S, Mizuno Y, Molina JA, Mukaetova-Ladinska EB, Pasquier F, Perry RH, Schulz JB, Trojanowski JQ, Yamada M (2005) Diagnosis and management of dementia with Lewy bodies: third report of the DLB Consortium. Neurology 65:1863–1872. doi: 10.1212/01.WNL.0000187889.17253.B1

5. McKeith IG, Galasko D, Kosaka K, Perry EK, Dickson DW, Hansen LA, Salmon DP, Lowe J, Mirra SS, Byrne EJ, Lennox G, Quinn NP, Edwardson JA, Ince PG, Bergeron C, Burns A, Miller BL, Lovestone S, Collerton D, Jansen ENH, Ballard C, De Vos RAI, Wilcock GK, Jellinger KA, Perry RH (1996) Consensus guidelines for the clinical and pathologic diagnosis of dementia with Lewy bodies (DLB): report of the consortium on DLB international workshop. Neurology 47:1113–1124. doi: 10.1212/WNL.47.5.1113

6. Raunio A, Kaivola K, Tuimala J, Kero M, Oinas M, Polvikoski T, Paetau A, Tienari P, Myllykangas L (2019) Lewy-related pathology exhibits two anatomically and genetically distinct progression patterns: a population-based study of Finns aged 85. Acta Neuropathol 138:771–782. doi: 10.1007/s00401-019-02071-3

7. Tanei Z ichi, Saito Y, Ito S, Matsubara T, Motoda A, Yamazaki M, Sakashita Y, Kawakami I, Ikemura M, Tanaka S, Sengoku R, Arai T, Murayama S (2021) Lewy pathology of the esophagus correlates with the progression of Lewy body disease: a Japanese cohort study of autopsy cases. Acta Neuropathol 141:25. doi: 10.1007/S00401-020-02233-8

8. Tanskanen M, Peuralinna T, Polvikoski T, Notkola IL, Sulkava R, Hardy J, Singleton A, Kiuru-Enari S, Paetau A, Tienari PJ, Myllykangas L (2008) Senile systemic amyloidosis affects 25% of the very aged and associates with genetic variation in alpha2-macroglobulin and tau: a population-based autopsy study. Ann Med 40:232–239. doi: 10.1080/07853890701842988
